# Long-term results of irrigated bipolar radiofrequency ablation in patients with recurrent arrhythmia after failed unipolar ablation

**DOI:** 10.1097/MD.0000000000019970

**Published:** 2020-05-22

**Authors:** Artur Baszko, Karol Kochman, Tomasz Królak, Piotr Kałmucki, Wojciech Telec, Stefan Ożegowski, Andrzej Szyszka

**Affiliations:** a Department of Cardiology, Poznań University of Medical Sciences; b Department of Cardiology, Medical University of Gdańsk, Poland.

**Keywords:** active and ground electrodes, bipolar radiofrequency ablation, scar formation, ventricular tachycardia

## Abstract

**Introduction:**

The RF ablation of ventricular tachycardia (VT) or atrial flutter (AFl) can be unsuccessful due to lack of lesion transmurality. Bipolar ablation (BA) is more successful than unipolar ablation (UA). The purpose of our study was to investigate the long-term effect of BA ablation in patients after failed UA.

**Methods:**

Patients with septal VT (5) or AFL (2) after 2 to 5 unsuccessful UA were prospectively analysed after BA. All patients presented with heart failure or had ICD interventions.

**Results:**

BA was successful in 5 patients (1 failure each in the AFL and VT group). The follow-up duration was 10 to 26 months. In AFL group, BA was successful in 1 patient, unidirectional cavotricuspid block in was achieved in the other patient. All patients were asymptomatic for 12 months, but 1 had atrial fibrillation and the other had AFL reablation 19 months after BA. In VT group, all patients had several forms of septal VT. BA was successful in 4 patients. In 2 patients with high septal VT BA resulted in complete atrioventricular block. During follow-up, 1 patient had VT recurrence 26 months after BA and died after an unsuccessful reablation. Three patients had VT recurrences of different morphologies, which required reablation (UA in 2 and alcohol septal ablation in the other patient).

**Conclusion:**

BA was successful in patients with AFL and septal VT resistant to standard ablation. Relapses of clinical arrhythmia are rare; however, long-term follow-up is complicated by recurrences of different arrhythmias related to complex arrhythmogenic substrate

## 1 Introduction

Recurrent drug refractory tachyarrhythmia is a well-recognized indication for radiofrequency (RF) ablation.^[1]^ The electroanatomical systems with contact force measurements not only improve our understanding of the arrhythmogenic substrate but also help in obtaining durable transmural scar.^[2]^ However, even high energy RF ablation fails to interrupt re-entry that is responsible for tachycardia, when the circuit is located deeply within the myocardium.^[3,4]^ Recently, bipolar ablation (BA) was proposed as a safe and effective method for eliminating focal or re-entrant form of arrhythmia.^[5,6]^ In this situation, the RF current flows between 2 ablation electrodes located on the opposite sides of the tachycardia circuit.^[7]^ The experimental studies tested various configurations of electrode types, positions with respect to the tissue, RF energy, and application duration in order to set the optimal parameters of ablation. The limited number of clinical reports show favorable effects obtained with different ablation parameters. In all cases, BA was attempted after several failed unipolar procedures.^[8]^ The increased knowledge on early and late outcomes may influence our decisions to consider BA on earlier stages of the procedure. We aimed to investigate the long-term outcome of patients who have recurrent atrial and ventricular reentrant tachycardias after a failed unipolar ablation (UA), which were treated with bipolar ablation.

## 2 Methods

Patients with recurrent or incessant, highly symptomatic atrial flutter, or ventricular tachycardia after failed classical UA were prospectively included in our study. The study protocol was approved by our local ethic committee and all patients gave written informed consent. The bipolar RF ablation was performed using 2 electrodes located on the opposite sites of the arrhythmogenic substrates. The active electrode was 3.5-mm tip irrigated SmartTouch or NaviStar Thermocool (Biosense Webster, Diamond Bar, CA) connected to an RF generator (Stockert). The ground electrode was a non-irrigated electrode with a 4-mm or 8-mm tip (7F Celsius, Biosense Webster, Diamond Bar, CA) or irrigated tip electrode (ThermoCool SF) connected to the indifferent electrode receptacle of the RF generator using a custom-made connector. The detailed description of the method has been published elsewhere.^[6,9]^

### 2.1 Atrial flutter group

**Figure 1:**
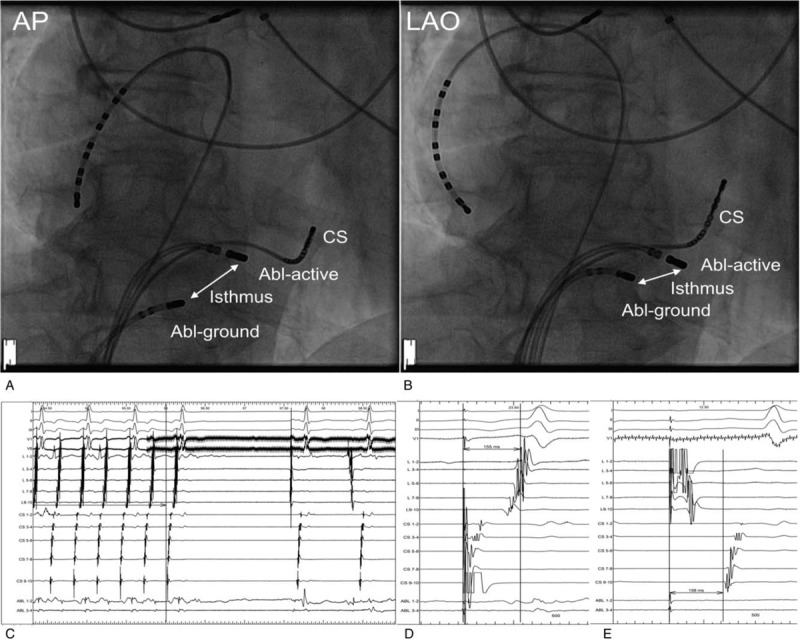
Bipolar ablation of the cavo-tricuspid isthmus dependent atrial flutter after failed ablation. (A) Antero-posterior projection during ablation. (B) Left oblique projection during ablation. The decapolar electrodes are positioned in coronary sinus (CS) and at the lateral wall of the right atrium. The active ablation electrode (Abl-active) is positioned just behind the tricuspid valvular ring. The ground electrode (Abl-ground) is located at the most proximal part of the isthmus. Before ablation the Abl-ground electrode is connected to the electrophysiological system to assess the morphology of the signal and check the impedance to avoid delivering the energy while electrode is in the inferior venae cavae. (C) The intracardiac signals at the time the successful bipolar application was delivered. During the application the Abl-ground electrode is connected to the indifferent electrode receptacle of the RF generator, thus the intracardiac signals are not visible. (D) Pacing form proximal CS electrodes shows block within the cavo-tricuspid isthmus. (E) Pacing from distal decapolar electrodes at the lateral wall confirms the bidirectional block.

After obtaining femoral access, the electrodes were positioned within coronary sinus (deflectable 10 pole 5F, Irvine Biomedical, St. Jude Medical, CA) and within right atrium (4 pole 6F, St. Jude Medical, DeVeau Place, MN). The entrainment was used to confirm that arrhythmia is dependent on the cavo-tricuspid isthmus. When the CARTO system (Biosense Webster, Diamond Bar, CA) was used, the detailed map was created to identify the possible sites of conduction. In all cases, BA was preceded with linear RF application between the tricuspid valve and inferior vena cava with an irrigated ablation electrode (25 ml/min saline flow, 40 W). If the flutter was not interrupted, BA was performed. The 8-mm tip electrode was connected to the electrophysiological system and RF generator to record the signals and the impedance. The electrode was positioned on the most proximal portion of the isthmus where the small atrial potential was still present and the impedance was within normal limits. This was performed to avoid delivering the energy within the vein, as the high impedance could prevent the delivery of application. The position of the electrode was documented using X-ray in LAO 30° and RAO 30° projections. Thereafter, the active irrigated tip electrode was reconnected to the CARTO system and positioned in the most distal segment of the isthmus, where the largest ventricular potential was recorded with a small atrial component. The RF energy was set to 30 W with 25-ml/second saline flow. The application was delivered for 90 seconds or until flutter was terminated. If the application did not terminate the tachycardia, the active electrode was moved back to record the larger atrial potential, and application was delivered again. The example of cavo-tricuspid isthmus bipolar ablation is presented in Figure 1.

### 2.2 The VT group

**Figure 2:**
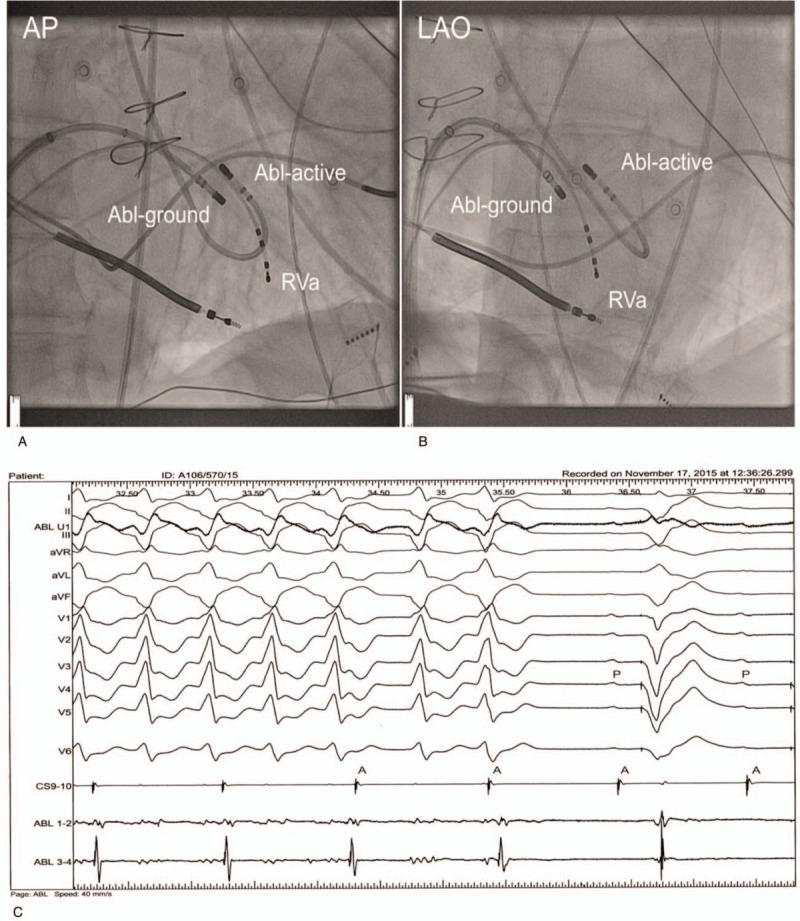
The example of septal VT treated with the bipolar ablation. (A) Antero-posterior projection. (B) Left anterior oblique projection. The ablation electrode (Abl-active) was introduced retrogradely through aortic valve and positioned on the superior aspect of the interventricular septum. The ground electrode (Abl-ground) was positioned on the opposite (right) site of the septum. The SL0 sheath was been used for mapping of the left ventricle, thereafter was stabilizing the ground electrode during ablation. (C) The ablation of the incessant ventricular tachycardia. After termination of the tachycardia the advanced atrioventricular block was present. Pacing was delivered by the ICD. After decreasing the rate of pacing the third degree of block was confirmed.

All patients with VT scheduled for BA had documented arrhythmia related to interventricular septum. Diagnostic electrodes were positioned as described above. In cases of incessant VT, the attempt was performed to terminate it with overdrive pacing. Programmed VT stimulation was performed at the beginning of the study with 1 to 3 premature complexes. All morphologies of VT were recorded. The electroanatomical map with the CARTO 3 system was performed using the SmartTouch electrode of the right and left ventricles and the aortic root. Fragmented and double potentials were marked. In the ventricular endocardium, areas with a bipolar voltage of >1.5 mV were considered normal. Those with a bipolar voltage of <0.5 mV were areas of dense scare, whereas areas with a voltage of 0.5 to 1.5 mV were the border zones between the scar and the normal myocardial tissue.^[10]^ The pace-mapping was performed from both sides of the septum to obtain the sites with the best match between clinical VT and paced QRS complexes. This was important, because the previous applications resulted in the areas of very low potentials that could not be interpreted clearly. The sites with the best match from the left and right sites were marked. Information from potential maps and pace-mapping was taken to plan the position of BA. BA was preceded by a standard UA. If this was unsuccessful, BA was performed. The 4-mm tip electrode was connected to the electrophysiological system and positioned on the previously marked best sites at the right ventricle septum. Thereafter, the 3.5-mm irrigated tip active ablation electrode (SmartTouch) was positioned at the best site of the left-sided septum. The position and the distance between the electrodes were measured using fluoroscopy and CARTO system in several projections. The RF application was continued for 90 seconds; then, the electrode was moved to the next point in an attempt to create the linear lesion according to the previously marked points. In cases of incessant tachycardia, when the arrhythmia was terminated during application, the site was marked, but the application was continued for 90 seconds. After the linear application was completed, VT stimulation was performed using up to 4 premature complexes. When the clinical tachycardia was still induced, the passive electrode was slightly moved and ablation was repeated. The end point was non-inducibility of the clinical VT. The example of cavo-tricuspid isthmus bipolar ablation is presented in Figure 2.

### 2.3 Follow-up

Patients were followed in the outpatient clinics. Patients after flutter ablation underwent 24-hour electrocardiography (ECG) unless a pacemaker was implanted. The AFL episode longer than 30 seconds was considered a recurrence. Patients with VT ablation had ICD/CRT-D implanted with home monitoring function. Any VT faster than 120/min and lasting for more than 15 seconds or requiring ICD intervention was considered a recurrence of arrhythmia.

## 3 Results

From November 2015 to February 2018, 9 patients were included in our study. A total of 7 patients underwent BA. In 2 patients with incessant atrial flutter, repeat UA was successful; hence, BA was not necessary. In the patients who underwent BA, 2 patients had typical, cavo-tricuspid atrial flutter, whereas 5 patients had septal-type VT.

### 3.1 Atrial flutter group

Two female patients (aged 59 and 69 years) with incessant atrial flutter and symptoms of heart failure due to arrhythmic cardiomyopathy underwent BA (Table 1). The cycle length of AFL was 270 ms and 276 ms, respectively, with 2:1 conduction in both cases. In 1 patient, CARTO 3 was used, whereas in 1 patient ablation was performed with the classical approach. The CARTO 3 map was created using a 3.5-mm bidirectional Thermocool Navistar electrode. The propagation map showed a slow conduction region within the central part of the isthmus. The entrainment form at central site of the isthmus revealed post-pacing interval equal to flutter cycle length. The 8-mm tip Celsius was used as the ground electrode and was positioned at the most proximal part of the isthmus. In this patient, the single 90-second application was delivered and terminated the atrial flutter in 10 seconds with evidence of bidirectional block. Afterwards, another 60-second application was delivered. The patient in whom standard X-ray-guided ablation was performed required several applications as the flutter was only terminated after 100 seconds of BA, but there was still unidirectional conduction through the isthmus. Finally, after 4 applications, a significant conduction delay was achieved, without a bidirectional block. This procedure was considered a partial success. The total RF duration was 450 seconds.

**Table 1:**
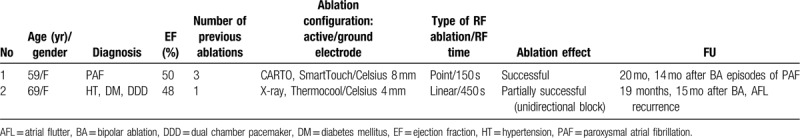
Clinical and procedural characteristics of bipolar radiofrequency ablation in patients with typical atrial flutter.

The mean procedure duration was 82.5 minute (range 75–90 minute), fluoroscopy time was 19 minute (range 8–30 minute), and X-ray dose was 74.5 mGy (range 29–120 mGy).

### 3.2 VT group

The VT group consisted of 5 male patients (mean age: 69 years, range 56–74 years) with ischemic (n = 2), non-ischemic (n = 2), and hypertrophic cardiomyopathy (HCM) (n = 1). The characteristics of the group are presented in Table 2. The mean ejection fraction of patients was 33.4% (range 12–55%). The clinical details of the patients are represented in Table 2. During EPS, 2 septal VTs were induced in 3 patients, 3 VT forms in 2, and 4 VT forms in 1 patient. The patient with HCM also had spontaneous left ventricular summit VT. The CARTO 3 map of the left ventricle presented a small region of high septal or subaortic scaring of 23 cm^2^ (range 6–98 cm^2^). On the basis of the ECG morphology and detailed mapping, the location of the VT was considered in the high septum in 3 patients, in the low septum in 1 patient, and in the high to medium septum in 1 patient. The active ablation electrode was SmartTouch in all patients, whereas the ground electrode was 4-mm tip Celsius in 4 patients and ThermoCool SF in 1 patient.

**Table 2:**
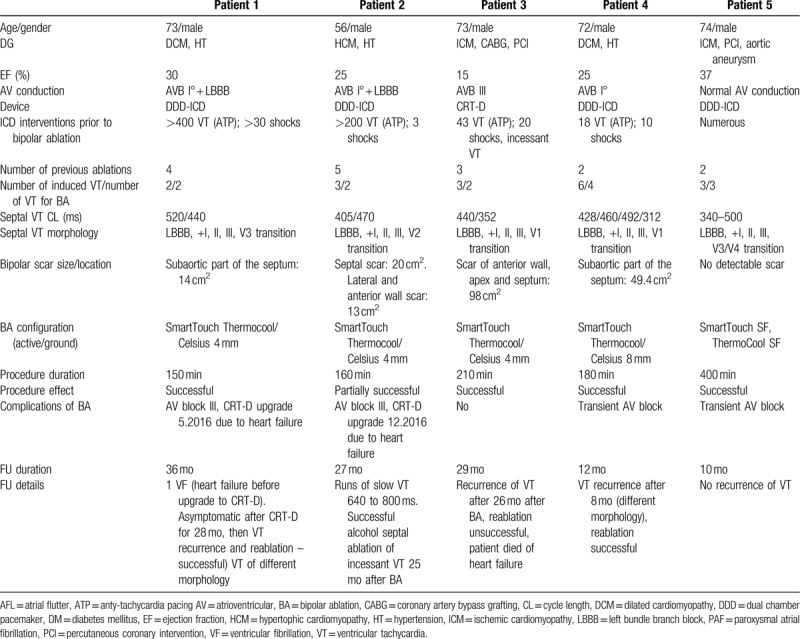
Clinical and procedural characteristics of bipolar radiofrequency ablation in patients with septal ventricular tachycardia (VT).

The VT ablation was performed during VT in 3 patients resulting in VT termination after a mean BA duration of 34.5 seconds (range 16–53 seconds). The application was performed in the high septum in 2 patients and in the low septum in one. Remaining patients had linear bipolar RF ablation (high septum in 1 patient and low septum in 1). The mean duration of BA was 1630 seconds, ranging from 1160 seconds to 2100 seconds.

After ablation, VT stimulation was performed with up to 4 extra stimuli from the left and right septum. In 4 patients, no VT was inducible, whereas in 1 patient VF was induced and terminated with defibrillation. Ablation of LV summit VT was unsuccessful in the patient with HCM (spontaneous runs of VT).

The complete atrioventricular block occurred in 2 patients who underwent high septal VT ablation. One of these patients had left bundle branch block (LBBB) caused by previous sequential UA (HCM), whereas the other patient had a block occurring after VT was terminated with RF application. In the third patient, there was prolongation of AV conduction during application, which returned to normal within 30 seconds. In 2 patients with more distal locations of septal VT, no complications were observed. The mean procedure duration was 212 minutes (range 110–400 minutes), fluoroscopy time was 34 minutes (range 15–57 minutes), and X-ray dose was 103.6 mGy (range 41–206 mGy).

### 3.3 Follow-up

#### 3.3.1 Atrial flutter group

The follow-up duration of patients after bipolar cavo-tricuspid ablation was 19 and 20 months. Patients received oral anticoagulants without antiarrhythmic drugs. After ablation, the symptoms of heart failure improved with normalization of LV function in both patients. One patient with a dual chamber pacemaker (DDD) pacemaker (Etrinsa DR, Biotronik) implanted before ablation had no arrhythmia for 15 months, but experienced several episodes of typical flutter. The other patient did not present any clinical arrhythmia, but after 14 months, the patient experienced several episodes of atrial fibrillation.

#### 3.3.2 VT group

The mean follow-up duration was 23 months (range 10–36 months). Antiarrhythmic treatment with amiodarone was continued in 3 patients. Detailed information on patients is presented in Table 2. During follow-up, 1 patient with ICM and CRT-D died 26 months after BA. The patient had VT recurrence 24 months after index ablation; underwent 2 ablations, which were unsuccessful; and died due to progression of heart failure. The morphology of the recurrent VT was different from the initially treated arrhythmia. Two patients who experienced AV block as a consequence of BA required an upgrade of ICD to CRT-D because of the symptoms of heart failure. One patient with DCM and post-ablation AV block had VF treated with high energy shock during progression of heart failure before undergoing CRT-D. After 28 months, the patient had a recurrence of clinical arrhythmia and underwent additional ablation, which was successful (no VT at the 3-month follow-up). The patient with HCM had asymptomatic short episodes of slow VT (90–110/min), which did not require ICD intervention (monitoring zone) of septal origin (no LV summit VT despite an initially unsuccessful ablation), but the patient had incessant VT thereafter, which was successfully treated with ethanol injection to the septal artery. The third patient with DCM and ICD was free from VT for 14 months but had several episodes of fast VT of a different morphology, underwent reablation, and was asymptomatic for the next 6 months. The last patient had no VT recurrence at the 10-month follow-up.

## 4 Discussion

Our study showed that BA was successful in 5 out of 7 patients with AFL or VT after several failed UA. All patients were free from clinical arrhythmia for 12 months thereafter. During long-term follow-up most of patients experienced the recurrence of arrhythmia but mainly of different morphology and reablation was in most cases successful.

It has been established that absence of VT recurrence after ablation is associated with improved survival.^[11,12]^ However, in a substantial proportion of patients, the ablation is unsuccessful or the VT recurs. In the collaborative study published by Tung et al, 33% of patients with structural heart disease had VT still inducible after ablation. The recurrence rate of VT was 30%. Patients who died had 55% recurrence rate of VT compared to 22% in those who survived.^[11]^ The MRI study performed after failed VT ablation revealed that the significant proportion of patients had an intramural scar pattern.^[13]^ The identification of the intramural substrate with electroanatomical systems may not be sufficient, and standard RF energy applied sequentially from opposite sites may not be effective. UA has a limited efficacy in cases of arrhythmia located in the interventricular septum, middle layer of the myocardium, or in papillary muscles.^[14]^ It has been well documented that scar formation during RF current delivery is related to the electrode size, the energy power, the contact force between the electrode and the tissue, and the cooling effect of surrounding fluid, and only a small part of the energy is delivered to the tissue.^[15]^ The high energy bipolar RF ablation can result in deeper lesions when current flows between 2 electrodes located on the opposite sites of the myocardium, improving the success rate of the procedure.^[16]^ Nguyen et al recently investigated the impact of electrode tip size, irrigation, and its orientation with respect to the tissue.^[14]^ The 2 irrigated electrodes orientated perpendicularly created the largest and deepest lesions. We have also documented that there is an optimal configuration of contact force and RF duration, and the use of an 8-mm tip ground electrode results in a high proportion of lesion transmurality in the swine heart model.^[17]^

The clinical application of BA was proved for focal arrhythmia arising from the LV summit, atrial flutter, and more complex substrates of electrical storms.^[9,18,19]^ These studies show that BA is acutely successful in cases of failed unipolar procedures. However, follow-up of patients with a complex arrhythmogenic substrate is not optimistic, as they experience relapses of clinical and additional arrhythmia. Koruth et al investigated patients with septal atrial flutter who had failed UA.^[9]^ In 3 patients, 5 forms of flutter with critical isthmus within the interatrial septum were documented. All flutters were terminated with short-lasting BA (16 ± 6 seconds). During a relatively short follow-up of 6 months, all patients had relapses of clinical flutter (2 required reablation) and 1 had relapses of flutter and fibrillation. In our group of patients with cavo-tricuspid atrial flutter, the long-term follow-up was more favorable as only patients with a partially successful procedure had arrhythmia recurrence more than 12 months after the procedure. The cavo-tricuspid isthmus has several anatomic variants that can create an obstacle to successful ablation: isthmus length, shape (straight, convex, or pouch-type), a prominent Eustachian ridge, or an extensive network of Chiari.^[20]^ The relatively well-described anatomical target for ablation with complex anatomy can be a good target for BA.

In the same paper by Koruth et al, in 6 patients with septal or free wall VT with multiple morphologies, the bipolar RF ablation was acutely successful in the majority of patients without complications.^[9]^ During the follow-up, 2 patients with septal VT had no recurrences, 1 had single VT terminated with antitachycardia pacing, and 1 had recurrences and required reablation, but finally died due to heart failure. One patient after successful ablation of free wall VT was arrhythmia free at the 12-month follow-up. The second patient with unsuccessful ablation required septal alcohol ablation. In our case series, the majority showed several forms of VT related to septal substrates. It is important to note that patients with non-ischemic septal substrate for VT have no or very small scars detected by the electroanatomical system. The similar observation was reported by Haqqani et al.^[3]^ The endocardial mapping with CARTO system revealed dense scar areas of 28 ± 23 cm^2^, which is very close to our findings. We should focus our attention on the typical ECG pattern of septal VTs (LBBB and normal or left axis deviation), especially those with high septal origin, and this may be important to guide our procedures.

Another important finding is the risk of atrioventricular block during BA of septal VT. The risk is high when the ablation is performed in high septal locations, and is not particularly related to the unipolar or bipolar type of ablation. Haqqani et al performed late enhanced MRI revealing the septal substrate in 31 patients undergoing repeated ablation.^[3]^ There was a high proportion of pre-existing atrioventricular conduction blocks (8 had complete atrioventricular block and 10 had LBBB). It is not stated whether this conduction abnormalities were related to underlying diseases or previous ablations. The ablation was performed from both sides of the interventricular septum and was successful in 66% of the patients, but new atrioventricular block occurred in another 5 patients. After a median follow-up of 12 months, 26% of the patients died and 16% underwent heart transplantation due to heart failure. In our study, the atrioventricular block occurred only when the applications were performed in high septal locations, and in all cases, the applications terminated the clinical VT.

The long-term follow-up after BA is not very optimistic, but the technique is used only in cases when the standard approach with UA has failed. Nevertheless, the concept of BA can be a very important adjunct to standard unipolar approach in cases when the critical part of the arrhythmia (focal or re-entry) is located deeply in the myocardium. There is a need for longer prospective study to validate the method's effectiveness and safety.

### 4.1 Study limitation

This is prospective analysis of non-randomized patients referred for repeated ablation after previous failed attempts. The late enhanced MRI would be of benefit to precisely identify and describe the potential substrate within the septum. The value of MRI could be limited by previously performed ablations from both sides of the interventricular septum and ICD implantation. In our study, the different ground electrodes were used. This was related to the observation that 8-mm tip or irrigated electrodes can result in a higher transmurality rate. The significant limitation is the lack of contact force recording from the ground electrode. The electroanatomical system could not also visualize the ground electrode during application, and the changes of its position were only documented by fluoroscopy. The numerous previous ablations performed in the cavo-tricuspid isthmus and on both sides of the interventricular septum created a scarring zone with low potentials and non-excitability with high energy pacing, which could influence the precision of the mapping. We believe that BA is applicable on earlier stages of the procedure together with contact force measurement, and visualization of the ground electrode could positively influence both acute and long-term success rates, but this needs to be proven in larger scale studies.

## 5 Conclusions

Bipolar RF with a standard and electroanatomic system can be used to terminate arrhythmias in selected patients with an intramural substrate. The acute success of BA is achievable in most patients; however, the long-term outcomes are not favorable as there is a high incidence of recurrences. The risk of atrioventricular block is high when the arrhythmogenic substrate is located high in the interventricular septum. Consideration of the BA procedure in earlier stages of treatment together with improvement in recording of parameters of ground electrodes can influence the long-term success rate.

## Author contributions

**Conceptualization:** Artur Baszko, Karol Kochman.

**Data curation:** Andrzej Szyszka.

**Formal analysis:** Artur Baszko.

**Investigation:** Artur Baszko, Karol Kochman, Piotr Kałmucki.

**Methodology:** Artur Baszko.

**Project administration:** Artur Baszko.

**Resources:** Artur Baszko, Tomasz Królak, Piotr Kałmucki, Wojciech Telec, Andrzej Szyszka.

**Software:** Wojciech Telec.

**Supervision:** Artur Baszko, Andrzej Szyszka.

**Writing – original draft:** Artur Baszko, Karol Kochman.

**Writing – review & editing:** Artur Baszko, Piotr Kałmucki, Wojciech Telec, Stefan Ożegowski, Andrzej Szyszka.

## References

[1] Priori SG Blomström-Lundqvist C Mazzanti A 2015 ESC guidelines for the management of patients with ventricular arrhythmias and the prevention of sudden cardiac death: the task force for the management of patients with ventricular arrhythmias and the prevention of sudden cardiac death of the European Society of Cardiology (ESC). Eur Heart J 2015;36:2793–867. 26745817 10.1093/eurheartj/ehv445

[2] Andreu D Berruezo A Ortiz-Pérez JT Integration of 3D electroanatomic maps and magnetic resonance scar characterization into the navigation system to guide ventricular tachycardia ablation: clinical perspective. Circ Arrhythm Electrophysiol 2011;4:674–83. 21880674 10.1161/CIRCEP.111.961946

[3] Haqqani HM Tschabrunn CM Tzou WS Isolated septal substrate for ventricular tachycardia in nonischemic dilated cardiomyopathy: incidence, characterization, and implications. Heart Rhythm 2011;8:1169–76. 21392586 10.1016/j.hrthm.2011.03.008

[4] Ikeda A Nakagawa H Lambert H Relationship between catheter contact force and radiofrequency lesion size and incidence of steam pop in the beating canine heart: electrogram amplitude, impedance, and electrode temperature are poor predictors of electrode-tissue contact force and lesion size. Circ Arrhythm Electrophysiol 2014;7:1174–80. 25381331 10.1161/CIRCEP.113.001094

[5] Piers SRD Dyrda K Tao Q Bipolar ablation of ventricular tachycardia in a patient after atrial switch operation for dextro-transposition of the great arteries. Circ Arrhythm Electrophysiol 2012;5:e38–40. 22511664 10.1161/CIRCEP.111.969345

[6] Futyma P Wysokińska A Sander J Bipolar endo-epicardial radiofrequency ablation of arrhythmia originating from the left ventricular summit. Circ J 2017;82:1721–2. 29046505 10.1253/circj.CJ-17-0782

[7] González-Suárez A Trujillo M Koruth J Radiofrequency cardiac ablation with catheters placed on opposing sides of the ventricular wall: computer modelling comparing bipolar and unipolar modes. Int J Hyperth 2014;30:372–84. 10.3109/02656736.2014.949878 25256891

[8] Sivagangabalan G Barry MA Huang K Bipolar ablation of the interventricular septum is more efficient at creating a transmural line than sequential unipolar ablation. PACE 2010;33:16–26. 20449877 10.1111/j.1540-8159.2009.02602.x

[9] Koruth JS Dukkipati S Miller MA Bipolar irrigated radiofrequency ablation: a therapeutic option for refractory intramural atrial and ventricular tachycardia circuits. Heart Rhythm 2012;9:1932–41. 22863684 10.1016/j.hrthm.2012.08.001

[10] Marchlinski FE Callans DJ Gottlieb CD Linear ablation lesions for control of unmappable ventricular tachycardia in patients with ischemic and nonischemic cardiomyopathy. Circulation 2000;101:1288–96. 10725289 10.1161/01.cir.101.11.1288

[11] Tung R Vaseghi M Frankel DS Freedom from recurrent ventricular tachycardia after catheter ablation is associated with improved survival in patients with structural heart disease: an International VT Ablation Center Collaborative Group study. Heart Rhythm 2015;12:1997–2007. 26031376 10.1016/j.hrthm.2015.05.036 PMC4549209

[12] Silberbauer J Oloriz T Maccabelli G Noninducibility and late potential abolition: a novel combined prognostic procedural end point for catheter ablation of postinfarction ventricular tachycardia. Circ Arrhythm Electrophysiol 2014;7:424–35. 24833642 10.1161/CIRCEP.113.001239

[13] Njeim M Yokokawa M Frank L Value of cardiac magnetic resonance imaging in patients with failed ablation procedures for ventricular tachycardia. J Cardiovasc Electrophysiol 2016;27:183–9. 26445386 10.1111/jce.12848

[14] Nguyen DT Tzou WS Brunnquell M Clinical and biophysical evaluation of variable bipolar configurations during radiofrequency ablation for treatment of ventricular arrhythmias. Heart Rhythm 2016;13:2161–71. 27424078 10.1016/j.hrthm.2016.07.011

[15] Wittkampf FHM Nakagawa H RF catheter ablation: lessons on lesions. Pacing Clin Electrophysiol 2006;29:1285–97. 17100685 10.1111/j.1540-8159.2006.00533.x

[16] Nagashima K Watanabe I Okumura Y Lesion formation by ventricular septal ablation with irrigated electrodes: comparison of bipolar and sequential unipolar ablation. Circ J 2011;75:565–70. 21187654 10.1253/circj.cj-10-0870

[17] Baszko A Telec W Kałmucki P Bipolar radiofrequency ablation: the impact of tip load, application duration, power, and indifferent electrode size on the transmurality of a lesion. Heart Beat J 2019;1:101–6. DOI 10.24255/hbj/102680.

[18] Baszko A Telec W Kałmucki P Bipolar irrigated radiofrequency ablation of resistant ventricular tachycardia with a septal intramural origin: the initial experience and a description of the method. Clin Case Rep 2016;4:957–61. 27761246 10.1002/ccr3.648 PMC5054470

[19] Sauer WH Steckman DA Zipse MM High-power bipolar ablation for incessant ventricular tachycardia utilizing a deep midmyocardial septal circuit. Heart Rhythm Case Rep 2015;1:397–400. 10.1016/j.hrcr.2015.01.018 PMC5419695 28491595

[20] Caldwell JC Hobson N Redfearn D Importance of anatomy in cavotricuspid isthmus. Europace 2016;18:950. 10.1093/europace/euv441 26857192

